# Amyloid‐Guided Assembly of Magnetically Recyclable Enzymatic Nanofibers Toward Sustainable Biocatalysis

**DOI:** 10.1002/advs.78579

**Published:** 2026-11-05

**Authors:** Shuangping Liu, Qilin Yang, Qian Zhang, Tiantian Liu, Xiao Han, Jian Mao

**Affiliations:** ^1^ State Key Laboratory of Food Science and Technology School of Food Science and Technology Jiangnan University Wuxi Jiangsu China; ^2^ Shaoxing Key Laboratory of Traditional Fermentation Food and Human Health Jiangnan University (Shaoxing) Industrial Technology Research Institute Shaoxing Zhejiang China; ^3^ National Engineering Research Center of *Huangjiu* Zhejiang Guyuelongshan Shaoxing Wine Co., Ltd Shaoxing Zhejiang China

**Keywords:** amyloid nanofibers, fusion‐enzyme engineering, magnetic biocatalysts, protein self‐assembly, sustainable biocatalysis

## Abstract

The rational construction of recyclable protein‐based catalytic materials requires connecting molecular enzyme design with higher‐order supramolecular organization and functional recovery interfaces. This study establishes a hierarchical design strategy that integrates fusion‐enzyme engineering, amyloid‐guided self‐assembly, and magnetic immobilization. The primary amine oxidase DPAO4 and the flavin‐containing amine oxidase DMAO7 were joined through flexible or rigid linkers, and the rigid (EAAAK)_3_ variant provided broad substrate coverage, greater tolerance to process‐relevant stresses, and lower interdomain fluctuations in molecular dynamics simulations. N‐terminal Sup35^1‐61^ or Ure2^1‐80^ domains promoted the formation of ThT‐responsive fibrillar assembly, and the resulting enzymatic nanofibers were coupled to amino‐functionalized Fe_3_O_4_@SiO_2_‐NH_2_ nanoparticles. The composites enabled magnetic separation within 30 s, catalyzed the degradation of eight biogenic amines under acidic, thermal, saline, and ethanol‐containing conditions, and retained an average of 53.2% of their initial activity after four recovery cycles. As proof‐of‐concept evaluation in complex matrices, selected systems removed up to 70.6% of total biogenic amines from fermented beverages and 69.7% from soy sauce. The distinct responses of the Sup35‐ and Ure2‐based composites suggest an association between assembly‐domain identity and catalytic performance in complex matrices. This work demonstrates a hierarchical design approach for recyclable protein‐based catalytic materials and sustainable biocatalysis.

## Graphical abstract

A hierarchical strategy integrates linker engineering, amyloid‐guided self‐assembly, and magnetic immobilization to construct recyclable DPAO4‐DMAO7 enzymatic nanofibers. The resulting magnetic composites enable biogenic amine conversion in complex food matrices and simulated wastewater, while rapid magnetic separation supports repeated reuse. The work demonstrates a coordinated route from molecular design to recyclable catalytic materials.

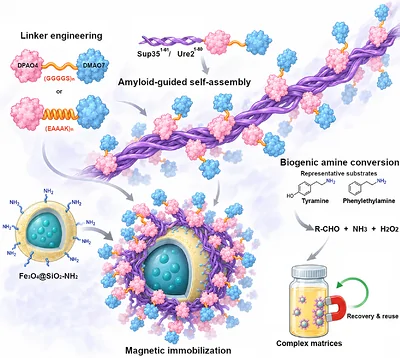

**Abbreviations**
BAs: Biogenic amines
CAD: Cadaverine
HIS: Histamine
PHE: Phenylethylamine
PUT: Putrescine
SPD: Spermidine
SPE: Spermine
TRY: Tryptamine
TYR: Tyramine

## 1 Introduction

Engineered protein‐based catalytic materials require molecular design decisions to be translated into higher‐order organization and macroscopic function. Free enzymes often lose activity during prolonged operation or exposure to acidic conditions, elevated temperatures, organic solvents, and high salinity [1, 2]. Conventional immobilization improves recovery, but it typically treats enzymes as passive cargo and offers limited control over catalytic‐domain spacing, orientation, and collective organization [2, 3, 4, 5, 6]. Surface crowding and uncontrolled loading can restrict active‐site access or perturb conformation. The central challenge is therefore not carrier selection alone, but a hierarchical design problem in which molecular architecture, supramolecular organization, and recovery must be coordinated across length scales.

Protein self‐assembly offers a route for propagating molecular information into functional biomaterial architectures. Biological systems organize catalytic proteins within spatially defined assemblies, including metabolon‐like structures that can increase local substrate availability and support functional cooperation [7]. Inspired by these principles, artificial enzyme assemblies have been developed for cascade catalysis, biosynthesis, and environmental applications [8, 9, 10, 11, 12]. Amyloid‐like protein fibrils are particularly attractive as genetically encodable scaffolds because they form stable, high‐aspect‐ratio nanostructures [13, 14, 15]. Amyloidogenic domains derived from the yeast prion proteins Sup35 and Ure2 can direct fusion proteins into fibrillar assemblies [13], while superparamagnetic particles can provide rapid separation and repeated recovery [16, 17]. These elements offer a basis for constructing functional protein materials, provided that catalytic function is preserved across each level of organization.

Bringing catalytic and assembly modules together nevertheless creates an interface problem: activity must be preserved as soluble enzymes are converted into higher‐order structures. Fusion‐protein engineering allows catalytic domains, assembly modules, and functional tags to be encoded within a single construct [18, 19], but performance depends strongly on domain connectivity. Inappropriate linkers can introduce steric interference, interdomain collapse, or restricted access to catalytic regions [20, 21, 22]. Flexible linkers provide conformational freedom, whereas rigid alpha‐helical linkers such as (EAAAK)_n_ can maintain domain separation and support independent folding [18, 19, 20]. These considerations motivate a three‐level design sequence comprising molecular engineering of catalytic‐domain connectivity, amyloid‐guided supramolecular organization, and coupling to a functional recovery interface.

This study demonstrates a hierarchical strategy for magnetically recyclable enzymatic nanofibers using the DPAO4‐DMAO7 system. Based on our previous characterization of *Saccharopolyspora hirsuta* T14, PAO4 and MAO7 were identified as key biogenic‐amine‐degrading enzymes with distinct catalytic profiles; their codon‐optimized derivatives, DPAO4 and DMAO7, were therefore combined with the aim of broadening substrate coverage [23]. The two enzymes were first connected through flexible or rigid linkers, after which Sup35^1‐61^ or Ure2^1‐80^ was introduced to promote fibrillar self‐assembly. The resulting protein nanofibers were coupled to amino‐functionalized Fe_3_O_4_@SiO_2_‐NH_2_ nanoparticles to create a magnetic recovery interface. Biogenic amine degradation was used as a proof‐of‐concept reaction to evaluate catalytic breadth, operation under acidic, thermal, saline, and ethanol‐containing conditions, repeated use, and performance in fermented foods and simulated wastewater. The study therefore examines how molecular engineering, protein self‐assembly, and materials integration can be coordinated within one catalytic system, while the transferability of this design to other enzyme combinations remains to be established.

## 2 Results and Discussion

### 2.1 Molecular Linker Engineering Defines an Assembly‐Compatible Catalytic Precursor

At the molecular‐engineering level, based on our previous characterization of *Saccharopolyspora hirsuta* T14, DPAO4 and DMAO7, the codon‐optimized derivatives of PAO4 and MAO7, were selected to combine their distinct biogenic‐amine‐degrading profiles within a bifunctional catalytic precursor [23]. The two enzymes were connected through flexible (GGGGS)_n_ or rigid (EAAAK)_n_ linkers of different lengths (Figures S1 and S2; Figure 1a,b). This common fusion‐enzyme framework enabled systematic comparison of linker‐dependent expression, catalytic breadth, and compatibility with subsequently introduced amyloidogenic domains. Induction conditions were adjusted to improve soluble expression of the approximately 124 kDa fusion proteins [18, 19, 20, 21, 24].

**FIGURE 1:**
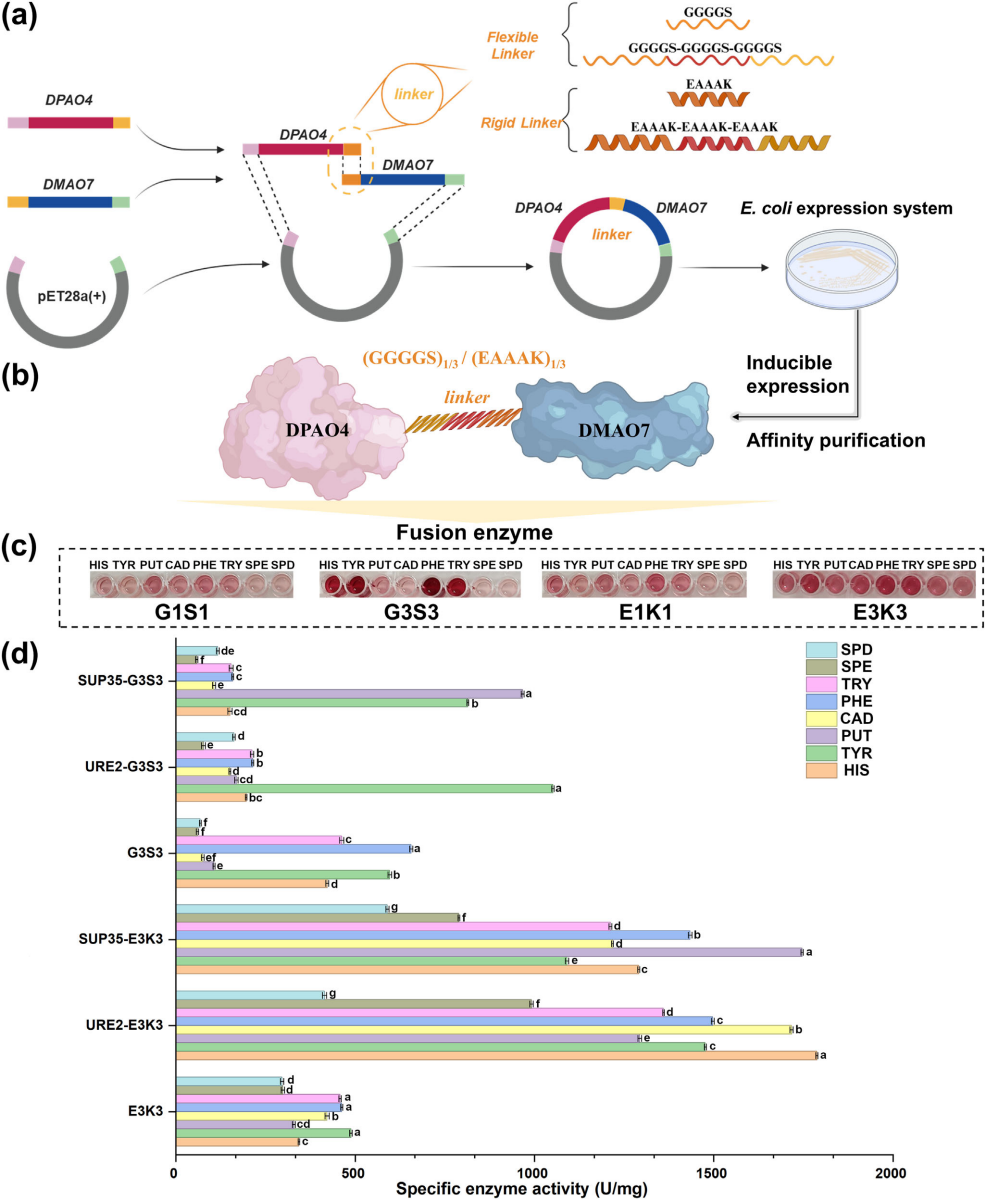
Linker engineering and catalytic evaluation of DPAO4‐DMAO7 fusion enzymes. (a) Construction of DPAO4‐DMAO7 fusion enzymes using flexible (GGGGS)_n_ or rigid (EAAAK)_n_ linkers. (b) Schematic representation of the linker‐connected DPAO4‐DMAO7 fusion enzyme. (c) Representative HRP‐coupled colorimetric substrate‐activity profiles of G1S1, G3S3, E1K1, and E3K3 toward eight biogenic amines. HIS, histamine; TYR, tyramine; PUT, putrescine; CAD, cadaverine; PHE, phenylethylamine; TRY, tryptamine; SPE, spermine; and SPD, spermidine. (d) Specific activities of G3S3 and E3K3 before and after N‐terminal fusion with Sup35^1‐61^ or Ure2^1‐80^ toward eight biogenic amines. Data in (d) are presented as mean ± SD (n = 3). Statistical significance was evaluated by one‐way ANOVA followed by Tukey's multiple comparisons test. Within each construct, different lowercase letters indicate significant differences among substrates (*p* < 0.05).

Linker architecture influenced soluble expression and catalytic performance. G1S1 and G3S3 required low‐temperature induction at 16°C to achieve maximal soluble expression, whereas E3K3 retained high catalytic activity after induction at 37°C (Figure S3). These observations are consistent with different folding or interdomain constraints in the flexible‐ and rigid‐linker constructs, although the expression data alone do not identify the underlying structural cause.

Catalytic activity was evaluated against eight representative biogenic amines, including histamine (HIS), tyramine (TYR), putrescine (PUT), cadaverine (CAD), phenylethylamine (PHE), tryptamine (TRY), spermine (SPE), and spermidine (SPD). Enzyme activity was quantified using an HRP‐coupled colorimetric assay based on H_2_O_2_ generation during amine oxidation, with absorbance monitored at 510 nm. Substrate‐activity profiling showed that linker design also changed biogenic‐amine coverage (Figure 1c). G3S3 showed higher activity toward selected monoamines, particularly histamine and tyramine, but lower activity toward the tested diamines. E3K3 produced a more balanced profile. For putrescine and cadaverine, E3K3 reached specific activities of 328.73 and 421.48 U mg^−1^, respectively, compared with 106.04 and 74.75 U mg^−1^ for G3S3 (Figure 1d). The data demonstrate a linker‐dependent change in catalytic breadth; possible structural differences between the constructs were further explored by modeling and molecular dynamics simulations.

Compatibility with the amyloidogenic domains was also evaluated by N‐terminal fusion of Sup35^1‐61^ or Ure2^1‐80^ to G3S3 and E3K3. The E3K3‐derived constructs showed higher activities than the corresponding G3S3‐derived constructs across most tested biogenic amines (Figure 1d). Together with the initial substrate‐activity profiling, these results identified E3K3 as a promising candidate. However, all four linker variants were further evaluated under process‐relevant conditions in Section 2.2 before final selection.

### 2.2 Linker‐Defined Architecture Establishes a Process‐Relevant Catalytic Operating Window

Catalytic properties of G1S1, G3S3, E1K1, and E3K3 were evaluated under conditions relevant to downstream application. For G1S1, E1K1, and E3K3, the optimal catalytic activities of most substrates were observed within the acidic range of pH 4.0‐6.0, whereas G3S3 showed greater substrate‐dependent variation across the tested pH range (Figure S4). pH‐stability measurements showed that G1S1, E1K1, and E3K3 generally retained higher residual activity after exposure to neutral to mildly alkaline conditions, whereas G3S3 displayed a more substrate‐dependent stability profile (Figure S5). The optimal pH and pH‐stability measurements describe different properties: the former reflects catalytic activity measured directly at a given pH, whereas the latter reflects residual activity after exposure to different pH conditions. Thus, an acidic optimum for catalytic activity can coexist with greater stability under neutral to mildly alkaline conditions [25, 26].

Temperature profiles showed a related linker‐dependent pattern (Figures S6 and S7). G3S3 showed a marked decline in activity at elevated temperatures, whereas E1K1 and E3K3 reached maximal activity at 55°C and retained substantial residual activity at 60°C. The higher‐temperature activity of the rigid‐linker constructs is consistent with reduced conformational freedom, but the present measurements do not directly resolve the mechanism [18, 19, 20, 21, 22]. Relative to reported amine‐oxidase and multicopper‐oxidase systems, the rigid‐linker variants operated across a favorable temperature window [27, 28].

Kinetic analysis further showed that linker choice changed catalytic efficiency, substrate coverage, and inhibition profiles (Table S1). G3S3 achieved high efficiency toward selected monoamines; its *k_cat_*/*K_m_* for tryptamine was 731.88 s^−1^ mM^−1^, approximately 43 times that of G1S1. E3K3 showed a more balanced kinetic profile across the tested substrates. Relative to E1K1, E3K3 showed catalytic efficiencies approximately 3.4 and 2.0 times those of E1K1 toward phenylethylamine and putrescine, respectively. Its *K_i_* for tyramine was 53.35 mM, compared with 12.64 mM for G3S3. These results indicate a trade‐off between high catalytic efficiency toward selected substrates and broader performance across the substrate panel.

Ethanol affected the catalytic activities of the linker variants in a construct‐ and substrate‐dependent manner (Figure S8). G1S1, E1K1, and E3K3 generally showed higher relative activities at elevated ethanol concentrations than under ethanol‐free conditions, whereas G3S3 exhibited more variable responses across the substrate panel. E3K3 maintained high relative activity toward the tested biogenic amines at 25% (v/v) ethanol.

Taken together, E3K3 provided the best overall balance of acidic‐pH activity, thermal tolerance, substrate coverage, inhibition resistance, and ethanol tolerance among the tested variants. Although flexible‐linker constructs showed higher activity toward selected substrates, E3K3 displayed a more balanced performance across the evaluated conditions. Accordingly, E3K3 was selected as the preferred linker architecture for subsequent assembly and magnetic immobilization studies.

### 2.3 Structural Modeling and Molecular Dynamics Contextualize Linker‐Dependent Catalytic Behavior

Three‐dimensional structures of DPAO4 and DMAO7 were predicted using AlphaFold2 [29] and evaluated with SAVES v6.0 and Ramachandran plots. More than 88% of DPAO4 residues and 91% of DMAO7 residues were in the most favored regions (Figure S9), supporting the use of these models for exploratory interface analysis while not substituting for an experimentally determined fusion‐enzyme structure [30].

HDOCK was used to evaluate a possible DPAO4‐DMAO7 interface [31]. The selected model had a Z‐score of −2.06, and PDBePISA analysis [32] gave a buried surface area of 1619.4 Å^2^ and an interaction energy of −13.4 kcal mol^−1^. These values describe one computationally plausible interdomain arrangement rather than a directly observed interface (Figure 2a). Within this model, predicted contacts included a salt bridge between DPAO4 Arg366 and DMAO7 Glu24 at approximately 3.0 Å, together with hydrogen bonds involving Arg416‐Ala86, Thr590‐Asp81, and Pro593‐Trp192 (Figure 2a). These contacts provide hypotheses for how the domains could interact, but their formation in the expressed fusion proteins remains to be experimentally verified [32, 33, 34].

**FIGURE 2:**
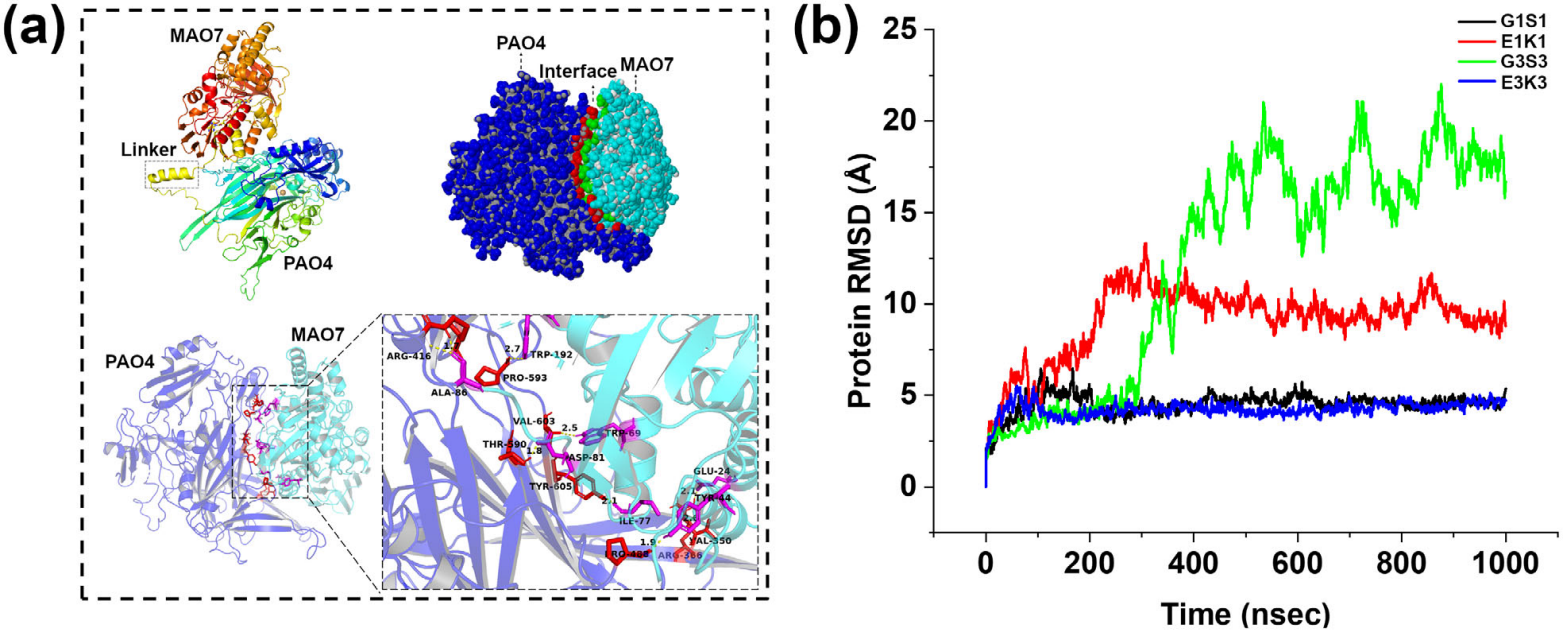
Structural modeling and molecular dynamics analysis of DPAO4‐DMAO7 fusion enzymes. (a) Predicted structural organization and DPAO4‐DMAO7 interaction interface in the fusion enzyme, including the linker‐connected model, predicted domain interface, and representative interfacial interactions. (b) RMSD trajectories of G1S1, G3S3, E1K1, and E3K3 during molecular dynamics simulations.

Molecular dynamics simulations further distinguished the linker‐dependent dynamics (Figure 2b and Figure S10). The flexible (GGGGS)_3_ construct showed large relative domain motion and RMSD values above 15 Å, whereas the rigid (EAAAK)_3_ construct reached a lower‐fluctuation state near 4 Å. These trajectories are consistent with lower interdomain fluctuations for E3K3 under the simulated conditions. However, the relationship between these simulated dynamics and the experimentally observed substrate profile remains correlative. Because the predicted interface was not experimentally validated and substrate‐entry pathways or catalytic events were not directly simulated, these calculations provide structural hypotheses rather than a mechanistic explanation for the broader catalytic profile of E3K3.

### 2.4 Amyloidogenic‐Domain Fusion Alters Catalytic Performance

The amyloidogenic domains Sup35^1‐61^ and Ure2^1‐80^ were fused to the N‐termini of G3S3 and E3K3 by overlap PCR. All four constructs were expressed in *Escherichia coli* BL21(DE3) at 16°C and purified as bands of approximately 135 kDa. Activity assays showed that N‐terminal fusion of the amyloidogenic domains altered the catalytic profile in a linker‐dependent manner (Figure 1d). Kinetic parameters for G3S3, URE2‐G3S3, SUP35‐G3S3, and E3K3 are summarized in Table 1; pH and temperature profiles are provided in Figures S11–S14, and solvent and salt tolerance in Figures S15 and S16. These data demonstrate construct‐dependent activity changes following amyloidogenic‐domain fusion. However, the present experiments do not establish whether these changes arise directly from fibrillar assembly or from fusion‐associated effects on protein folding, solubility, aggregation state, or assay‐related factors.

**TABLE 1:** Kinetic parameters of G3S3, URE2‐G3S3, SUP35‐G3S3, and E3K3 toward eight biogenic amines.

Enzyme	Parameter	HIS	TYR	PUT	CAD	PHE	TRY	SPE	SPD
G3S3	*V*_max_	1.95 ± 0.19^b^	1.80 ± 0.18^b^	0.79 ± 0.08^cd^	4.69 ± 0.47^a^	2.02 ± 0.20^b^	1.12 ± 0.11^c^	0.46 ± 0.05^de^	0.14 ± 0.02^e^
	*K*_m_	1.00 ± 0.12^d^	0.04 ± 0.004^e^	16.03 ± 0.80^a^	13.91 ± 0.69^b^	0.06 ± 0.01^e^	0.03 ± 0.003^e^	8.44 ± 0.42^c^	0.88 ± 0.09^de^
	*K*_i_	ND	12.64 ± 0.35^d^	ND	4.69 ± 0.15^e^	171.60 ± 3.25^b^	74.08 ± 1.85^c^	408.00 ± 8.15^a^	ND
	*k*cat	32.23 ± 0.95^b^	29.69 ± 0.88^c^	13.06 ± 0.45^e^	77.34 ± 2.32^a^	33.38 ± 0.99^b^	18.47 ± 0.55^d^	7.63 ± 0.23^f^	2.27 ± 0.07^g^
	*k*cat/*K*_m_	32.23 ± 0.96^c^	742.16 ± 18.55^a^	0.81 ± 0.03^d^	5.56 ± 0.17^d^	562.95 ± 13.85^b^	731.88 ± 17.95^a^	0.90 ± 0.03^d^	2.59 ± 0.08^d^
URE2‐G3S3	*V*_max_	1.25 ± 0.13^ab^	1.42 ± 0.24^a^	ND	1.47 ± 0.47^a^	0.87 ± 0.07^bc^	0.72 ± 0.09^c^	ND	0.51 ± 0.05^c^
	*K*_m_	1.94 ± 0.22^b^	1.3 ± 0.17^b^	ND	3.60 ± 0.66^a^	0.11 ± 0.02^c^	0.02 ± 0.0019^c^	ND	0.03 ± 0.0013^c^
	*K*_i_	ND	ND	ND	ND	435.40 ± 12.35a	94.77 ± 12.37b	ND	ND
	*k*cat	15.15 ± 0.45^ab^	17.12 ± 0.51^a^	ND	17.76 ± 4.53^a^	10.56 ± 2.32^bc^	8.64 ± 3.26c	ND	ND
	*k*cat/*K*_m_	7.80 ± 0.23^cd^	13.17 ± 0.39^c^	ND	4.94 ± 0.15^cd^	100.88 ± 2.52^b^	446.02 ± 11.15^a^	ND	ND
SUP35‐G3S3	*V*_max_	0.86 ± 0.06^ab^	1.09 ± 0.13^a^	ND	0.97 ± 0.09^a^	0.59 ± 0.08^bc^	0.50 ± 0.05^c^	0.43 ± 0.04^d^	0.40 ± 0.04^c^
	*K*_m_	1.41 ± 0.18^b^	1.84 ± 0.22^b^	ND	2.91 ± 0.57^a^	0.16 ± 0.04^c^	0.12 ± 0.03^c^	0.16 ± 0.04^c^	0.03 ± 0.003^c^
	*K*_i_	ND	ND	ND	ND	574.40 ± 7.74^a^	87.91 ± 2.20^b^	ND	148.00 ± 2.96^c^
	*k*cat	11.15 ± 3.33^ab^	14.22 ± 2.43^a^	ND	12.56 ± 3.38^a^	7.73 ± 1.23^bc^	6.51 ± 1.19^c^	5.63 ± 0.67^d^	5.17 ± 0.95^d^
	*k*cat/*K*_m_	7.93 ± 0.24^cd^	7.71 ± 0.23^c^	ND	4.32 ± 0.13^cd^	50.04 ± 6.25^b^	56.87 ± 7.71^a^	35.08 ± 1.05^d^	195.81 ± 4.90^d^
E3K3	*V*_max_	0.22 ± 0.02^cd^	0.74 ± 0.06^b^	1.08 ± 0.11^a^	0.34 ± 0.03^c^	0.17 ± 0.02^d^	0.18 ± 0.02^d^	0.16 ± 0.02^d^	0.27 ± 0.03^cd^
	*K*_m_	0.50 ± 0.05^ef^	4.39 ± 0.22^b^	10.26 ± 0.51^a^	2.26 ± 0.11^c^	0.35 ± 0.04^ef^	0.89 ± 0.09^e^	0.16 ± 0.02^f^	1.48 ± 0.07^d^
	*K*_i_	ND	53.35 ± 1.32^c^	ND	ND	132.10 ± 3.15^a^	57.88 ± 1.42^b^	ND	ND
	*k*cat	3.59 ± 0.11^e^	12.24 ± 0.36^b^	17.81 ± 0.53^a^	5.62 ± 0.17^c^	2.73 ± 0.08^f^	2.91 ± 0.09^f^	2.62 ± 0.08^f^	4.44 ± 0.13^d^
	*k*cat/*K*_m_	7.24 ± 0.22^c^	2.79 ± 0.08^de^	1.74 ± 0.05^f^	2.49 ± 0.07^e^	7.86 ± 0.23^b^	3.27 ± 0.10^d^	16.22 ± 0.48^a^	3.01 ± 0.09^de^

E3K3‐derived constructs generally exhibited higher absolute activities after amyloidogenic‐domain fusion than the corresponding G3S3‐derived constructs. The specific activity of URE2‐E3K3 toward putrescine was 1293.46 U mg^−1^, approximately 3.9 times that of E3K3 (328.73 U mg^−1^), while its activities toward histamine and cadaverine reached 1787.64 and 1716.81 U mg^−^^1^, respectively. SUP35‐E3K3 also increased activity, particularly toward putrescine and spermidine, and exceeded URE2‐E3K3 for selected substrates (Figure 1d). These data show that the assembly modules were compatible with the rigid‐linker backbone and did not impose a uniform activity penalty.

The G3S3‐derived constructs showed substrate‐dependent responses. URE2‐G3S3 exhibited putrescine and cadaverine activities approximately 1.6 and 2.0 times those of G3S3, respectively, whereas its activities toward histamine and phenylethylamine decreased. SUP35‐G3S3 showed a distinct profile, with putrescine and tyramine activities approximately 9.1 and 1.4 times those of G3S3, respectively (Figure 1d) These results indicate that the catalytic consequences of introducing an amyloidogenic domain depend on both the linker architecture and the substrate.

The activity changes observed after Sup35^1‐61^ or Ure2^1‐80^ fusion may reflect fusion‐associated changes in protein folding, local conformational constraints, solubility or aggregation state, and the microenvironment surrounding the catalytic domains, all of which could influence substrate accessibility and catalytic performance. The more consistent activity enhancement in the E3K3‐derived constructs suggests that the rigid‐linker architecture may better tolerate the additional N‐terminal amyloidogenic domain, whereas the G3S3‐derived constructs showed a more variable response. However, the present data do not distinguish among these possibilities or establish that the observed activity enhancement arises directly from amyloid assembly.

Across both amyloidogenic domains, the E3K3 backbone supported higher and broader activity than the corresponding G3S3 backbone. These results support a design sequence in which catalytic‐domain connectivity is optimized before an assembly module is introduced, without assigning the activity differences to a single unmeasured structural mechanism [35, 36, 37].

Units: *K*_m_ and *K*_i_, mM; *V*_max_, µmol/(L·min); *k*_cat_, s^−1^; *k*_cat_/*K*_m_, L/(s·mmol). Within each enzyme and kinetic parameter, values with different lowercase superscript letters are significantly different according to one‐way ANOVA followed by Tukey's multiple‐comparison test (*p* < 0.05).

### 2.5 Assembly Kinetics and Morphology Support Fibrillar Protein Materials

Assembly was monitored using 8‐anilino‐1‐naphthalenesulfonic acid (ANS) and thioflavin T (ThT) fluorescence, together with macroscopic observation and TEM. ANS was used to monitor changes in exposed hydrophobic environments during protein assembly. All four variants showed a time‐dependent rise in ANS fluorescence followed by a plateau after approximately 72 h (Figure 3b–e). SUP35‐E3K3 increased from 183.55 to 719.59 over 168 h. The emission maximum also shifted to shorter wavelengths, as illustrated by URE2‐G3S3 shifting from 520 to 504 nm, consistent with changes in the hydrophobic environments sampled by ANS during assembly [38, 39].

**FIGURE 3:**
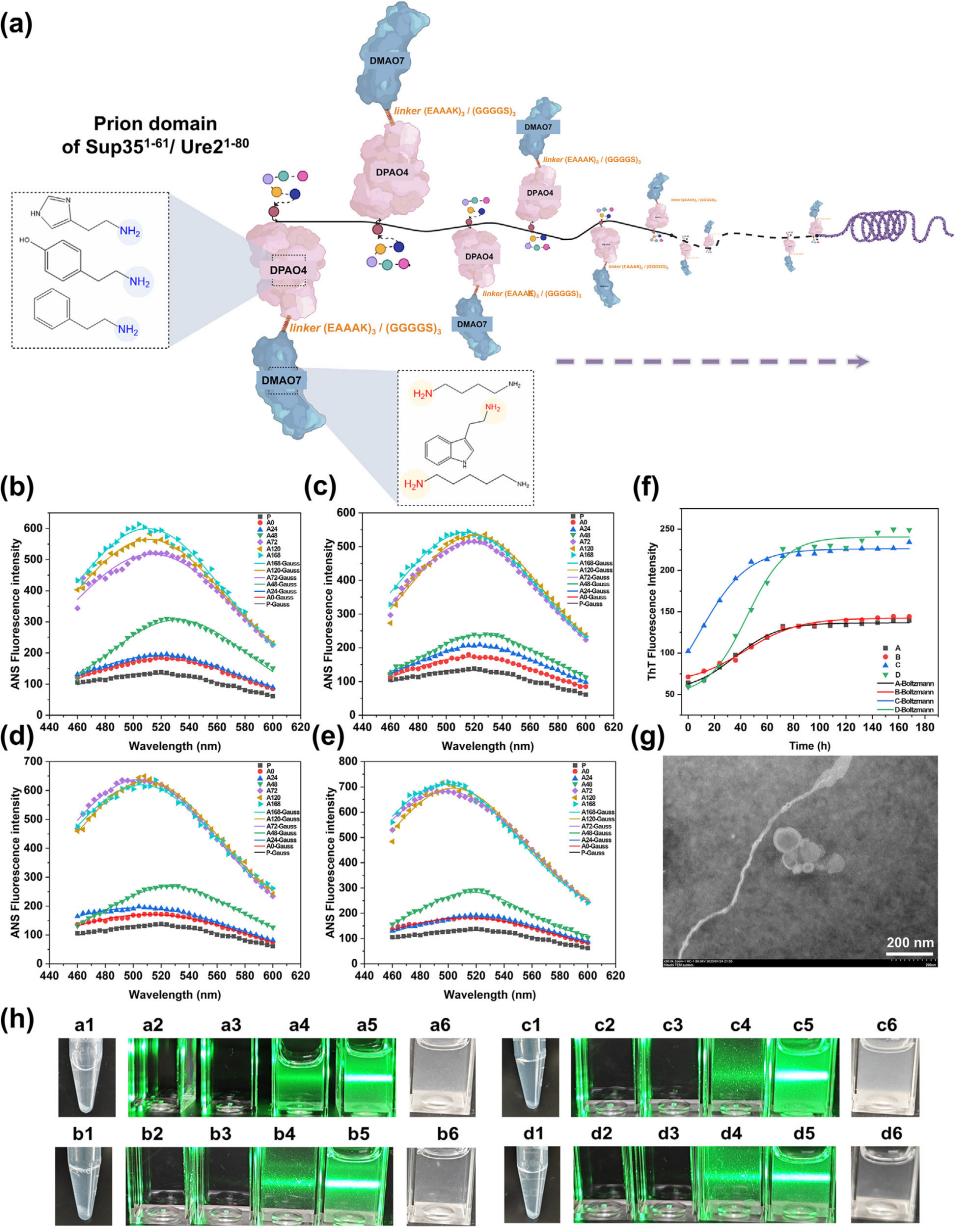
Amyloid‐guided assembly of fusion enzymes into fibrillar nanostructures. (a) Schematic representation of amyloidogenic‐domain‐mediated fibrillar assembly of the fusion enzymes. (b‐e) Time‐dependent ANS spectra for URE2‐G3S3, URE2‐E3K3, SUP35‐G3S3, and SUP35‐E3K3 over 168 h. (f) ThT fluorescence kinetics of the four constructs; curves A‐D correspond to URE2‐G3S3, URE2‐E3K3, SUP35‐G3S3, and SUP35‐E3K3. (g) Representative negative‐staining TEM image of the assembled fibrillar structures. (h) Macroscopic observation and Tyndall‐effect analysis of the four constructs. Images 1–6 correspond to natural sedimentation, assembly buffer, free enzyme, pre‐assembly nanozyme solution, assembled nanofibers under laser illumination, and assembled nanofibers without laser illumination, respectively. Labels a‐d correspond to URE2‐G3S3, URE2‐E3K3, SUP35‐G3S3, and SUP35‐E3K3, respectively.

ThT was used to monitor amyloid‐like fibrillar assembly. ThT fluorescence followed sigmoidal profiles with lag, growth, and plateau phases (Figure 3f). Boltzmann fitting gave lag times of 45.39‐84.04 h (Table S2), consistent with nucleation‐dependent assembly [40, 41, 42, 43, 44]. ANS and ThT report different aspects of assembly, and the fitted ThT lag times indicate construct‐dependent assembly kinetics. Sup35‐derived constructs reached higher final ThT intensities than the Ure2‐derived constructs. The fluorescence data support formation of ThT‐responsive assemblies but do not, on their own, define a unique fibril polymorph or catalytic‐domain arrangement [41].

Macroscopic light scattering and negative‐staining TEM provided complementary evidence for higher‐order assembly. The assembled samples showed a Tyndall effect (Figure 3h), and TEM revealed elongated, entangled fibrillar structures (Figure 3g). Together with the fluorescence kinetics, these observations support the conversion of the fusion proteins into fibrillar protein nanostructures. The measurements do not resolve whether the catalytic domains are uniformly displayed or remain properly folded within the fibrils. Accordingly, the present data demonstrate fibrillar assembly and construct‐dependent activity changes, but do not establish a causal relationship between them [35, 36, 37, 45, 46, 47].

### 2.6 Magnetic Immobilization Enables Recovery and Repeated Use

Different magnetic carriers were compared using protein loading, activity retention, and repeated‐use performance. Magnetic chitosan particles with diameters of 20 and 100 nm were evaluated alongside amino‐functionalized silica‐coated Fe_3_O_4_@SiO_2_‐NH_2_. Although 20 nm magnetic chitosan gave the highest loading, including up to 88.69% for Ure2‐derived constructs, nanofibers immobilized on Fe_3_O_4_@SiO_2_‐NH_2_ retained higher specific activity (Figure S17). Carrier selection was therefore based on functional retention rather than loading alone [16, 17, 48].

The Fe_3_O_4_@SiO_2_‐NH_2_ carrier combines a magnetic core, silica shell, and amino‐functionalized surface for glutaraldehyde‐mediated immobilization. This architecture is compatible with rapid magnetic handling and provides reactive sites for coupling [16, 17, 48, 49, 50, 51]. Under the tested conditions, Fe_3_O_4_@SiO_2_‐NH_2_ showed higher activity retention than the other carriers examined. However, because the carrier comparison simultaneously varies particle size, surface chemistry, and coupling environment, the present experiments do not establish the mechanistic basis of this difference. Silica‐coated magnetic particles have also been reported with amino‐functionalized, C18‐functionalized, and PVP‐modified surfaces [49, 50, 51].

Repeated‐use performance of Fe_3_O_4_@SiO_2_‐NH_2_‐immobilized nanofibers was evaluated over five reaction cycles, comprising the initial catalytic cycle followed by four magnetic recovery cycles (Figure 4b). Between consecutive cycles, the composites were magnetically separated and reused in the subsequent reaction. Activity in each cycle was normalized to that in the initial cycle. The relative activities, averaged across the four composites and eight substrates, were 100%, 93.86%, 69.66%, 61.19%, and 53.20% in cycles 1–5, respectively. Activity gradually declined during repeated operation. This decline cannot be attributed solely to enzyme inactivation, because protein desorption, material loss during recovery, or other changes during operation may also contribute. Fe_3_O_4_@SiO_2_‐NH_2_@URE2‐E3K3 showed the highest average residual activity in the fifth cycle (61.69%), while the relative performance of the other composites depended on the substrate.

**FIGURE 4:**
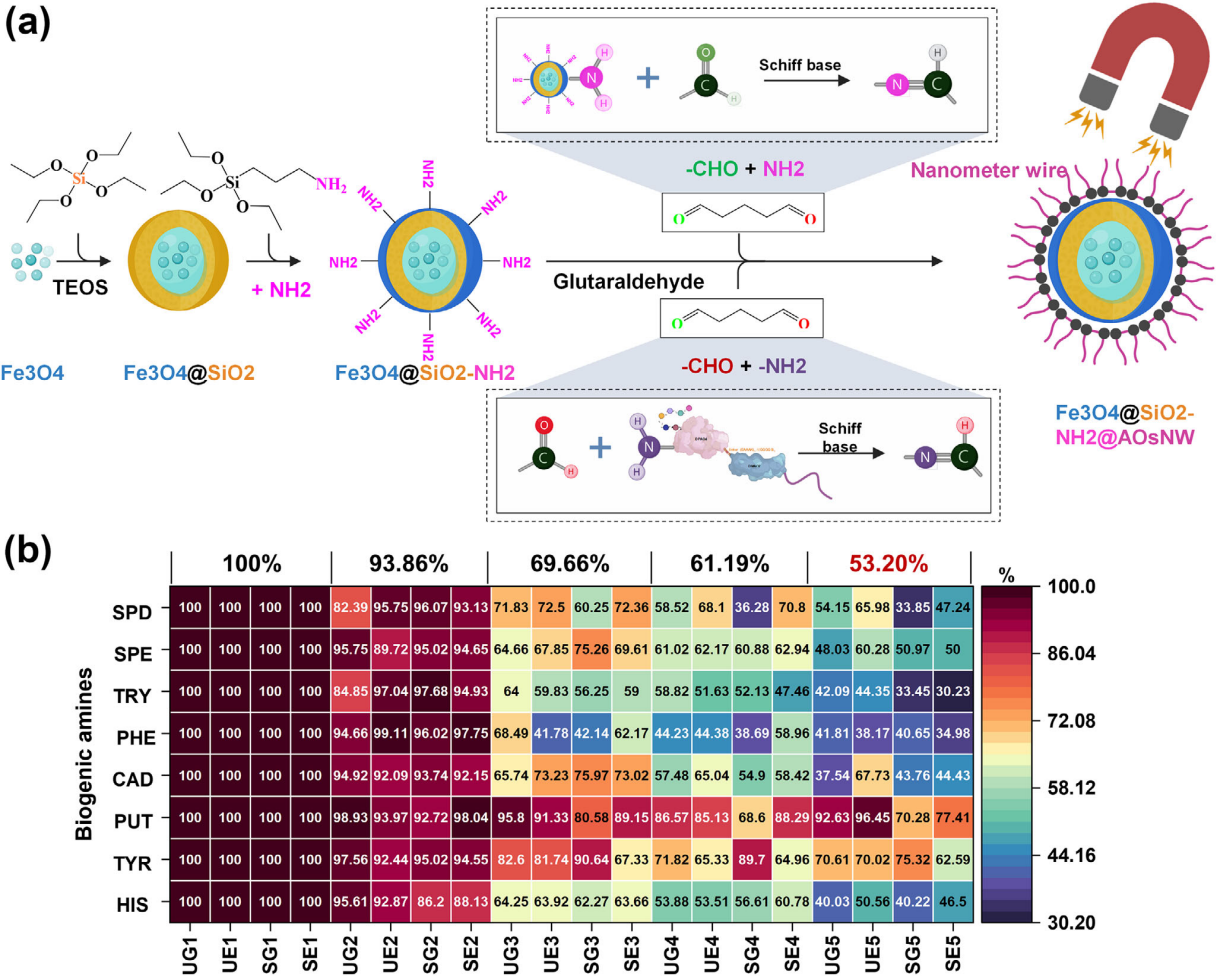
Magnetic immobilization and repeated use of amyloid‐guided enzymatic nanofibers. (a) Fabrication of the Fe_3_O_4_@SiO_2_‐NH_2_ carrier and glutaraldehyde‐mediated immobilization. (b) Relative catalytic activity of the immobilized nanofiber systems over five consecutive reaction cycles, including the initial cycle and four magnetic recovery cycles. Activity in the initial cycle was set to 100%, and residual activity was determined after each subsequent recovery cycle. UG, UE, SG, and SE denote Fe_3_O_4_@SiO_2_‐NH_2_@URE2‐G3S3, Fe_3_O_4_@SiO_2_‐NH_2_@URE2‐E3K3, Fe_3_O_4_@SiO_2_‐NH_2_@SUP35‐G3S3, and Fe_3_O_4_@SiO_2_‐NH_2_@SUP35‐E3K3, respectively; numbers 1–5 denote the corresponding reaction cycles.

### 2.7 Multimodal Characterization Verifies the Protein‐Inorganic Composite Interface

SEM images showed that Fe_3_O_4_@SiO_2_‐NH_2_ retained a quasi‐spherical particulate morphology after loading with URE2‐E3K3 or SUP35‐E3K3 nanofibers (Figure 5a–c). Severe particle aggregation was not evident in the imaged fields. Elemental mapping showed Fe, Si, and O from the carrier together with increased C and N signals after immobilization. Quantitative EDS analysis likewise showed higher organic‐element content in the composites (Table S3). These compositional changes are consistent with incorporation of protein‐containing material.

**FIGURE 5:**
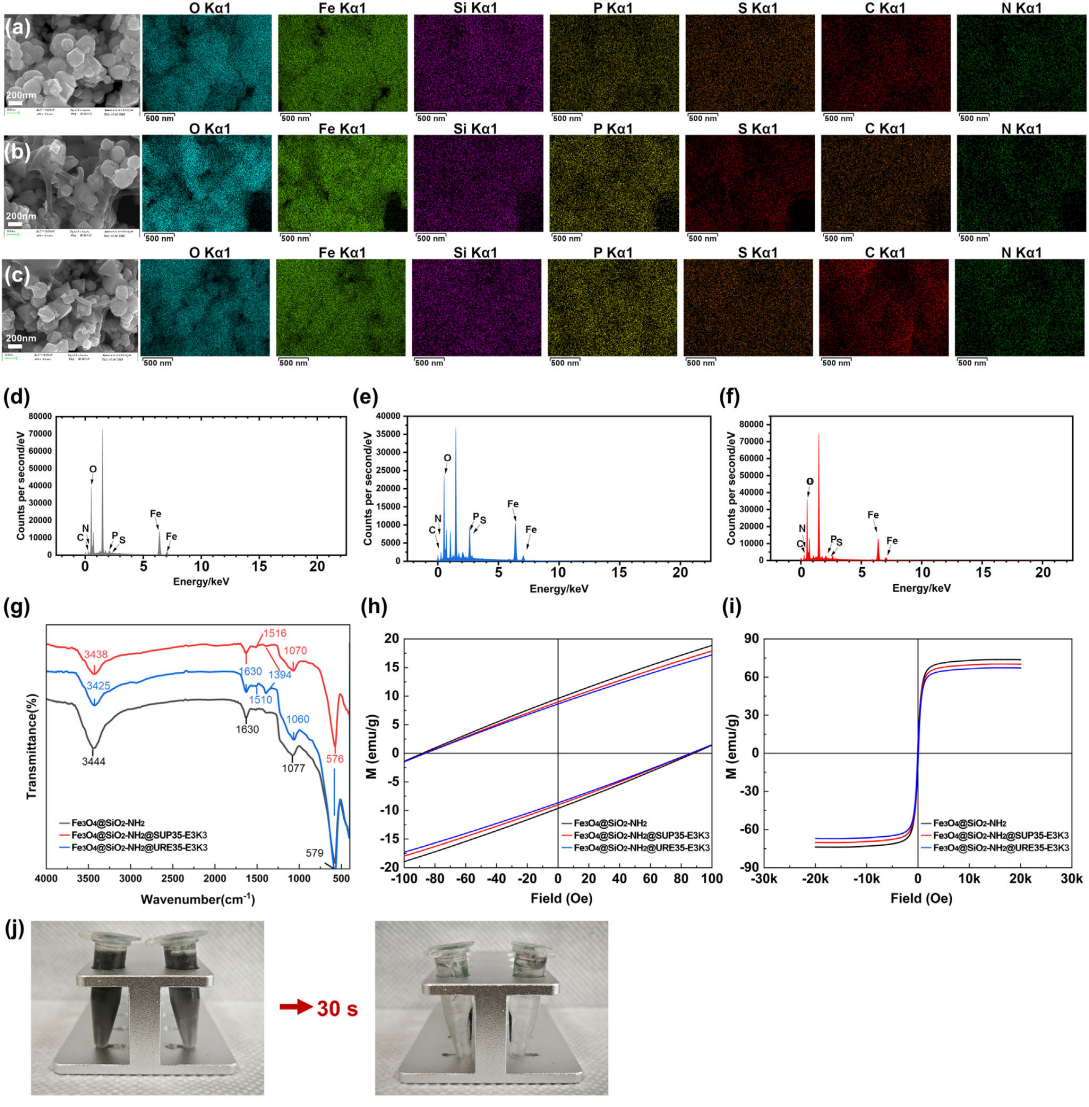
Physicochemical characterization of magnetically recyclable enzymatic nanofiber composites. (a‐c) SEM images and elemental maps of (a) Fe_3_O_4_@SiO_2_‐NH_2_, (b) Fe_3_O_4_@SiO_2_‐NH_2_@URE2‐E3K3, and (c) Fe_3_O_4_@SiO_2_‐NH_2_@SUP35‐E3K3. (d–f) Corresponding EDS spectra. (g) FT‐IR spectra. (h, i) Low‐field and full‐field magnetic hysteresis curves. (j) Magnetic separation within 30 s.

FT‐IR spectra provided additional evidence for protein incorporation and interfacial reaction with Fe_3_O_4_@SiO_2_‐NH_2_ (Figure 5g). The composites displayed amide I and amide II bands near 1630 and 1516 cm^−1^, respectively. Changes in aldehyde‐ and amine‐associated signals were consistent with glutaraldehyde‐mediated Schiff‐base formation. These spectra support coupling at the interface, while not excluding contributions from physical adsorption or other noncovalent interactions [17].

VSM analysis showed a modest decrease in saturation magnetization from 73.71 emu g^−1^ for Fe_3_O_4_@SiO_2_‐NH_2_ to approximately 67–70 emu g^−1^ for the nanofiber‐loaded composites (Figure 5h,i), consistent with addition of a nonmagnetic protein fraction. The composites retained superparamagnetic behavior and were separated from suspension within 30 s using an external magnet (Figure 5j) [48, 49, 50, 51].

SEM, EDS, FT‐IR, and VSM therefore provide complementary evidence for integration of the enzymatic nanofibers with the magnetic carrier across morphological, compositional, interfacial, and functional levels. The retained magnetic responsiveness links the supramolecular protein architecture to macroscopic recovery.

### 2.8 Hierarchical Integration Preserves Function Across Process‐Relevant Environments

Fe_3_O_4_@SiO_2_‐NH_2_@URE2‐E3K3 and Fe_3_O_4_@SiO_2_‐NH_2_@SUP35‐E3K3 were evaluated under pH, temperature, salinity, and ethanol conditions relevant to fermented foods and wastewater‐related environments (Figure 6). Their response profiles differed across the tested variables. These comparisons identify distinct operating windows for the two composites but do not separate the effects of assembly‐domain identity from possible differences in loading or nanofiber morphology [27, 28].

**FIGURE 6:**
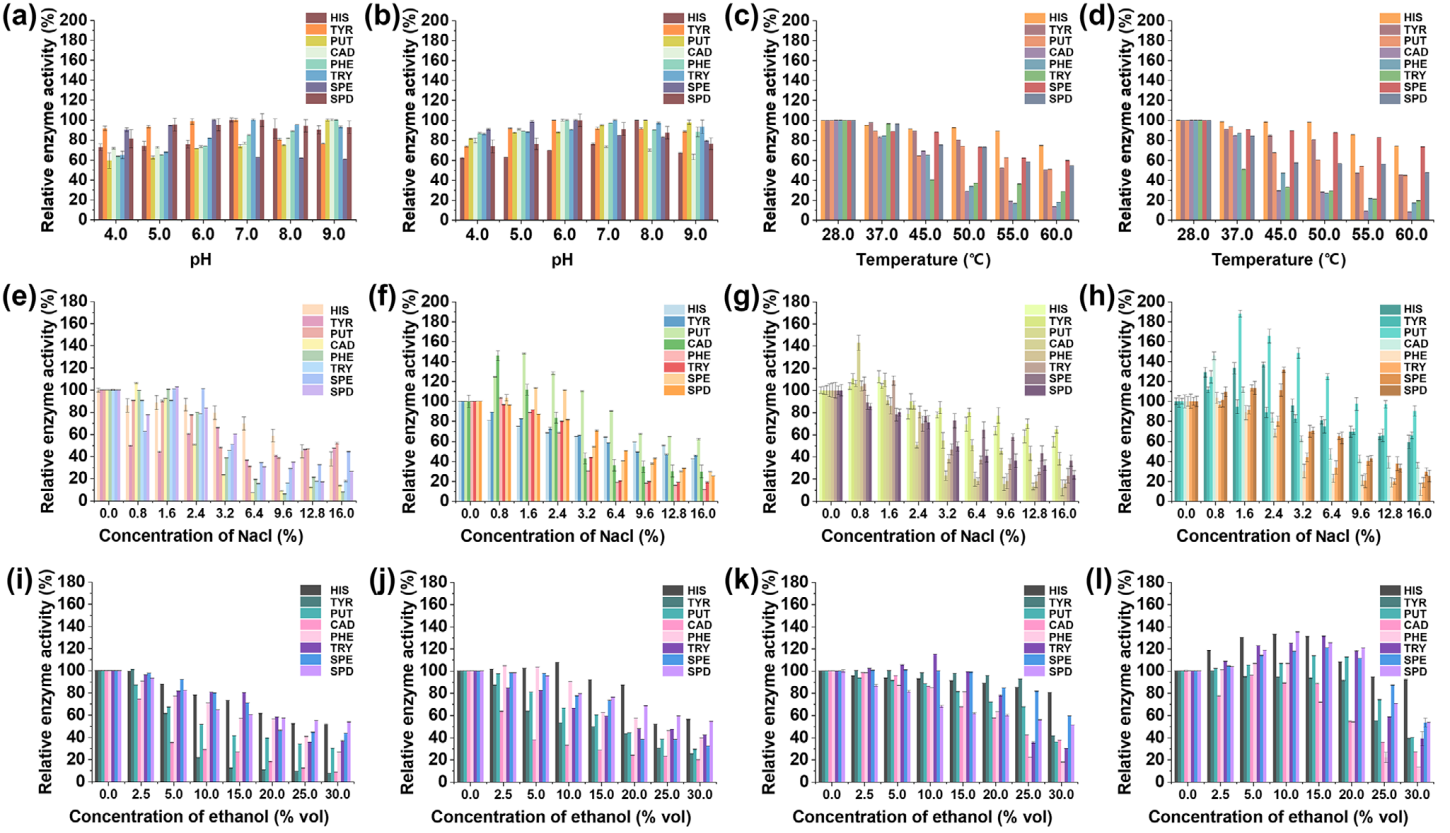
Catalytic performance of magnetically recyclable enzymatic nanofiber composites under process‐relevant stresses. (a, b) pH‐dependent activities of Fe_3_O_4_@SiO_2_‐NH_2_@URE2‐E3K3 and Fe_3_O_4_@SiO_2_‐NH_2_@SUP35‐E3K3. (c, d) Temperature‐dependent activities. (e–h) Effects of NaCl concentration on activity and residual stability. (i–l) Effects of ethanol concentration on activity and residual stability. Data are presented as mean ± SD (*n* = 3).

Fe_3_O_4_@SiO_2_‐NH_2_@URE2‐E3K3 showed favorable activity under acidic conditions, including high relative activity toward TYR and SPE at pH 5.0‐6.0 (Figure S18). Fe_3_O_4_@SiO_2_‐NH_2_@SUP35‐E3K3 performed better for several substrates near pH 7.0‐8.0. The different pH profiles show that assembly‐module selection is associated with different catalytic operating ranges after immobilization. Additional pH‐response measurements are shown in Figure 6a,b.

The composites showed substrate‐dependent temperature responses (Figure 6c,d and Figure S19). Temperature‐dependent catalytic activity and residual activity after thermal incubation describe different properties and should be interpreted separately.

Fe_3_O_4_@SiO_2_‐NH_2_@SUP35‐E3K3 also retained activity across a broad ethanol range (Figure 6i–l). Ethanol at 2.5%‐5.0% v/v enhanced activity toward selected substrates, and at 30% v/v the composite retained 56.45% activity toward HIS. Fe_3_O_4_@SiO_2_‐NH_2_@URE2‐E3K3 declined more strongly as ethanol concentration increased. The SUP35‐based composite was therefore the better‐performing system for the tested ethanol‐containing conditions.

Both composites remained active under NaCl stress (Figure 6e–h). The SUP35‐based composite showed an activation effect at low‐to‐moderate NaCl concentrations: PUT activity reached 148.07% at 1.6% NaCl. Measurable activity remained at the highest tested NaCl concentration, with substrate‐dependent retention. The URE2‐based composite was more sensitive to increasing salinity but remained catalytically active under high‐salt conditions.

The two assembly modules consequently generated complementary operating profiles. The SUP35‐based system performed better under ethanol, salinity, and elevated temperature for selected substrates, whereas the URE2‐based system showed favorable acidic‐pH performance. These observations associate assembly‐domain identity with matrix‐dependent performance within the present enzyme system; they do not establish a general rule for other enzyme combinations.

### 2.9 Proof‐of‐Concept Biogenic Amine Conversion in Complex Matrices

The two selected composites were evaluated in fermented foods and simulated wastewater, with biogenic amine concentrations determined by HPLC (Figure 7). Only the simulated wastewater was prepared with each of the eight target biogenic amines at 25 mg/L (200 mg/L total). Both reduced multiple biogenic amines in alcoholic fermented beverages. In *Huangjiu*, Fe_3_O_4_@SiO_2_‐NH_2_@SUP35‐E3K3 yielded 39.66 mg/L total biogenic amines compared with 91.38 mg/L in the carrier‐only control, corresponding to 56.60% lower total content, with complete removal of SPD and SPE. In red wine, the same composite achieved 70.60% total removal, with PHE and PUT removal above 92%. Removal in cooking wine was lower, 34.26%‐45.78%, under the combined matrix conditions (Figure 7b).

**FIGURE 7:**
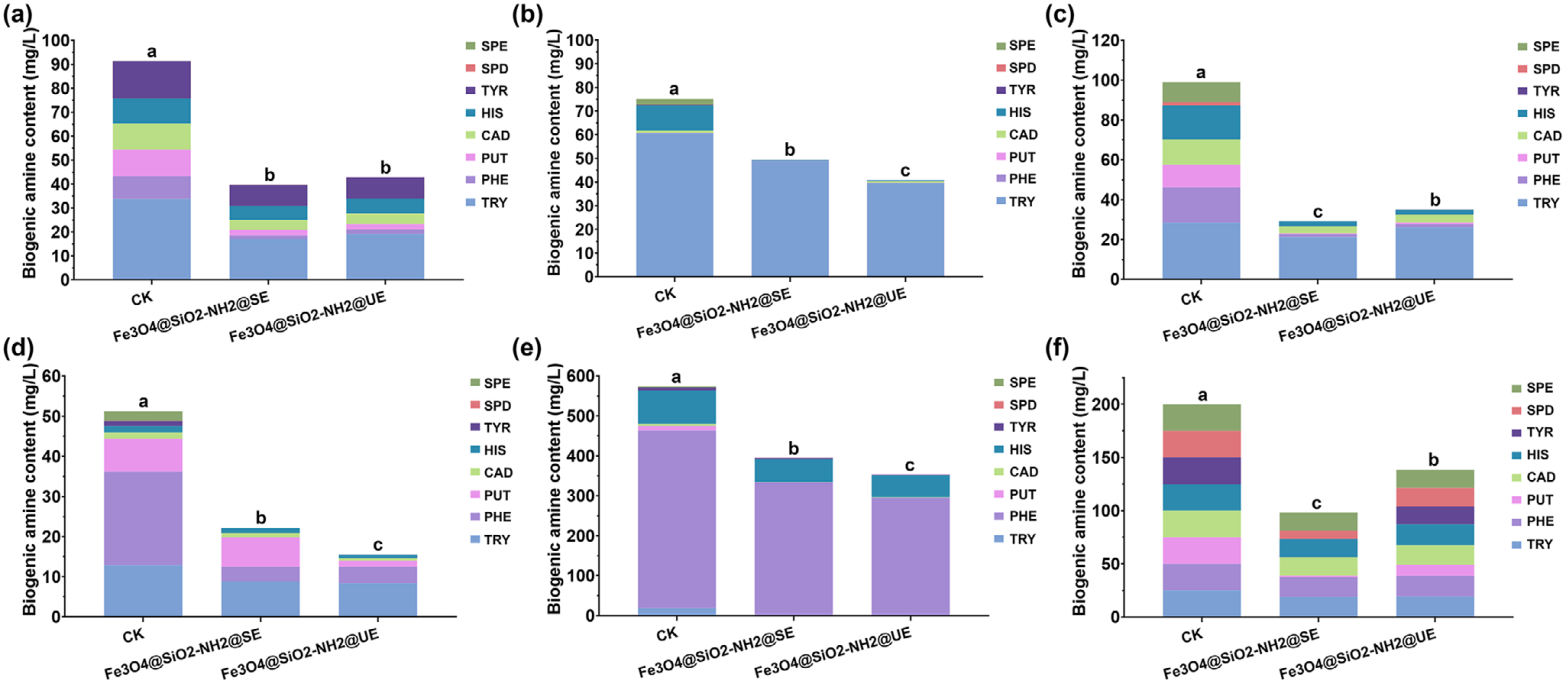
Biogenic amine removal in complex matrices. Biogenic amine contents in (a) *Huangjiu*, (b) cooking wine, (c) red wine, (d) soy sauce, (e) low‐salt soy sauce, and (f) simulated wastewater before and after treatment with Fe_3_O_4_@SiO_2_‐NH_2_@SUP35‐E3K3 (SE) or Fe_3_O_4_@SiO_2_‐NH_2_@URE2‐E3K3 (UE). CK denotes the carrier‐only control treated under the same conditions without immobilized enzyme, as described in Section 4.12. Within each matrix, total biogenic amine concentrations were compared among CK, SE, and UE using one‐way ANOVA followed by Tukey's multiple comparisons test. Groups that do not share a lowercase letter differ significantly at *p* < 0.05.

Performance differed in soy sauce (Figure 7d,e). In regular soy sauce, the URE2‐based composite outperformed the SUP35‐based system and achieved 69.74% total biogenic amine removal. In low‐salt soy sauce, percentage removal was approximately 31%‐38%, while the high starting concentration of 573.5 mg/L yielded a maximum removed amount of 220.53 mg/L. These results demonstrate matrix‐dependent performance without assigning the difference to a single physicochemical factor.

In the simulated biogenic‐amine‐containing wastewater, the bare magnetic carrier showed negligible removal, whereas Fe_3_O_4_@SiO_2_‐NH_2_@SUP35‐E3K3 removed 101.94 mg/L of total biogenic amines, including complete TYR removal and 93.52% PUT removal (Figure 7f). The negligible removal by the carrier control is consistent with an enzyme‐associated reduction in biogenic amine levels, although this control alone does not exclude adsorption by the protein‐containing composites.

These results provide proof‐of‐concept evidence that the hierarchical protein‐material system can reduce biogenic amine levels in fermented‐food and wastewater‐like matrices under the tested conditions. The two composites showed distinct performance profiles across acidic, saline, and ethanol‐containing environments, indicating that the choice of amyloidogenic domain can influence performance within the present system [25, 26, 52]. However, the present study evaluates biogenic amine conversion only and does not establish the safety of the treated food matrices. Because amine oxidation can generate H_2_O_2_ and corresponding aldehydes, product profiling and safety assessment will be required before practical food‐processing applications can be considered.

## 3 Conclusions

This study demonstrates a hierarchical design strategy for magnetically recyclable enzymatic nanofibers by integrating molecular engineering, amyloid‐guided supramolecular assembly, and a magnetic recovery interface. At the molecular level, the rigid (EAAAK)_3_ construct provided broad substrate coverage, favorable performance under process‐relevant stresses, and lower interdomain fluctuation in molecular dynamics simulations. At the assembly level, Sup35^1‐61^ and Ure2^1‐80^ promoted ThT‐responsive fibrillar organization. At the material level, coupling to Fe_3_O_4_@SiO_2_‐NH_2_ enabled separation within 30 s, and the composites retained an average of 53.2% of their initial activity after four magnetic recovery cycles. The systems remained active under acidic, thermal, saline, and ethanol‐containing conditions. As a proof‐of‐concept evaluation in complex matrices, selected composites removed up to 70.6% of total biogenic amines from fermented beverages and 69.7% from soy sauce. These experiments demonstrate biogenic amine conversion but do not establish food safety, which will require further product profiling and assessment of H_2_O_2_ and aldehyde formation. The distinct operating profiles of the Sup35‐ and Ure2‐based composites indicate that the choice of amyloidogenic domain can influence performance in complex matrices, although the physical basis of those differences remains to be resolved. The central contribution lies in integrating catalytic connectivity, amyloid‐guided self‐assembly, and magnetic recovery within a sequential hierarchical design, with the magnetic component serving as a practical recovery interface. This concept is demonstrated here in the DPAO4‐DMAO7 system, and its transferability to other enzyme systems remains to be established.

## 4 Experimental Section

### 4.1 Reagents

Commercial fermented food samples were sourced as follows: Huangjiu (18% ethanol, v/v) was obtained from Shaoxing, China; low‐salt soy sauce (8 g/100 mL NaCl), regular soy sauce (20 g/100 mL NaCl), cooking wine (589 mg/100 mL NaCl; ≥10% ethanol, v/v), red wine (≥12% ethanol, v/v), and fish sauce (933 mg/10 g NaCl) were purchased from a local market in Wuxi, Jiangsu Province, China. Luria‐Bertani (LB) and Terrific Broth (TB) culture media were acquired from Hope Bio‐Technology Co., Ltd. (Qingdao, China). All biogenic amine standards were purchased from Millipore Sigma (USA). All other chemicals and reagents were of analytical grade and obtained from Sinopharm Chemical Reagent Co., Ltd. (Beijing, China) or Shanghai Aladdin Biochemical Technology Co., Ltd. (Shanghai, China).

### 4.2 DNA Manipulation and Cloning

The PAO4 and MAO7 genes were codon‐optimized using Codon OptimWiz and the Codon Adaptation Index Calculator, designated as DPAO4 and DMAO7, and synthesized by Dynegene Technologies (Shanghai, China). Primers and linker sequences, including flexible linkers (GGGGS and (GGGGS)_3_) and rigid linkers (EAAAK and (EAAAK)_3_), are listed in Table S4. Fusion genes were constructed via Overlap Extension PCR (OE‐PCR). Briefly, the DPAO4 and DMAO7 fragments were individually amplified from their respective pET28a templates using specific primers (F1/R1 and F2/R2). After purification, the fragments were mixed in an equimolar ratio and subjected to a second round of PCR using overlap primers (OL‐F/OL‐R) to generate the full‐length fusion fragments. The resulting amplicons and the pET28a vector were digested with *Nhe* I and *EcoR* I. The purified fusion fragments were then cloned into the linearized vector using an In‐Fusion cloning kit (50°C for 15 min). The ligation products were transformed into competent *Escherichia coli* (*E. coli*) cells via heat shock. Positive transformants were screened by colony PCR using T7 promoter primers and subsequently verified by DNA sequencing. Correct clones were preserved at −80°C [53, 54].

### 4.3 Determination of Biogenic Amine‐Degrading Enzyme Activity and Protein Concentration

The enzymatic activity of amine oxidases was determined using a peroxidase‐coupled colorimetric assay based on the quantification of hydrogen peroxide (H_2_O_2_) generated during amine oxidation. In the presence of horseradish peroxidase (HRP), H_2_O_2_ reacts with 4‐aminoantipyrine and 2,4,6‐tribromo‐3‐hydroxybenzoic acid (TBHBA) to form a quinoneimine dye detectable at 510 nm. To evaluate substrate‐dependent activity, 10 mM solutions of various biogenic amines were used, including cadaverine (CAD), histamine (HIS), phenylethylamine (PHE), putrescine (PUT), tryptamine (TRY), tyramine (TYR), spermine (SPE), and spermidine (SPD). The reactions were conducted in 96‐well plates using a SpectraMax 190 Microplate Reader (Molecular Devices, USA). The 200 µL reaction system consisted of 10 µL enzyme solution, 20 µL substrate, 70 µL HRP (1.4 mg/mL), and 100 µL of working reagent (200 mM potassium phosphate buffer, pH 7.6, containing 1.5 mM 4‐aminoantipyrine and 1 mM TBHBA). Absorbance was monitored at 510 nm at 37°C for 10 min. One unit of enzyme activity (U) was defined as the amount of enzyme catalyzing the formation of 1 µmol of quinoneimine dye per minute under the assay conditions. Protein concentration was quantified using the Bradford method with bovine serum albumin (BSA) as the standard. All assays were performed in triplicate. To optimize protein expression, recombinant strains were induced at varying temperatures (16°C, 25°C, 28°C, and 37°C), and the optimal condition was determined based on the resulting enzyme activity [25, 54].

### 4.4 Characterization of Fusion‐Enzyme Catalytic Properties

The enzymatic properties of the fusion enzymes were systematically characterized across various conditions. To determine the optimal pH and temperature, enzyme activity was assayed over a pH range of 3.0–9.0 and a temperature range of 25°C–60°C, respectively. For pH and thermal stability assessments, the enzymes were pre‐incubated under the indicated conditions for a specified duration, after which the residual activity was measured. Kinetic parameters (*K_m_* and *V_max_*) were determined by measuring the initial reaction rates at varying substrate concentrations (0–30 mm) and fitting the data to the non‐linear Michaelis‐Menten equation [55]. Additionally, to evaluate the effect of organic solvents on catalytic performance, ethanol was added to the reaction system at final concentrations ranging from 0% to 25% (v/v), and the relative enzyme activity was quantified [25, 26, 27, 28].

### 4.5 Structural Modeling and Docking Analysis of DPAO4 and DMAO7

The three‐dimensional (3D) structures of DPAO4 and DMAO7 were predicted using AlphaFold2 [29]. To ensure structural reliability, the quality of the predicted models was evaluated using the SAVES v6.0 server. To investigate the protein‐protein interactions between the DPAO4 and DMAO7 domains, molecular docking was performed using the HDOCK online server. The interfacial properties of the docked complexes were subsequently analyzed using PDBePISA to quantify the interaction surface area and solvation energy [32, 33]. Finally, structural visualization and detailed interaction analyses were conducted using PyMOL [56].

### 4.6 Molecular Dynamics Simulations of Fusion‐Enzyme Variants

MD simulations of the fusion enzyme models were performed using the Desmond module within the Schrödinger software suite. The OPLS4 force field was employed to define intermolecular interactions, while water molecules were modeled using the SPC/E potential to balance computational accuracy and efficiency. Each protein complex was solvated in a cubic water box, and the system charge was neutralized by adding an appropriate number of sodium (Na^+^) or chloride (Cl^−^) ions. Prior to production runs, the systems underwent energy minimization to relax the initial geometry and eliminate steric clashes. The simulation was conducted under physiological conditions, maintaining the temperature at 310.15 K and pressure at 1.013 bar within the NPT ensemble. Finally, 100 ns unrestrained production simulations were carried out to capture the dynamic behavior of the complexes. Trajectory analysis, including RMSD, RMSF, hydrogen bond monitoring, and energy calculations, was performed using the Maestro interface [57].

### 4.7 Construction and Expression of Assembly‐Competent Fusion Enzymes

The gene fragments encoding the amyloidogenic domains Sup35^1‐61^ and Ure2^1‐80^ were amplified using specific primer sets (Sup‐F/R and Ure2‐F/R) designed with homologous arms to facilitate assembly [35, 36, 37]. Simultaneously, the pET‐28a(+) vectors containing the linker‐catalytic modules (G3S3 and E3K3) were linearized using primers OLPD‐F/R. Through homologous recombination, the amyloid domains were fused to the catalytic modules, yielding four recombinant expression vectors: pET‐28a(+)‐URE2‐G3S3, pET‐28a(+)‐URE2‐E3K3, pET‐28a(+)‐SUP35‐G3S3, and pET‐28a(+)‐SUP35‐E3K3. These constructs were initially transformed into *E. coli* DH5α for cloning and sequence verification. Upon confirmation, the plasmids were transformed into the expression host *E. coli* BL21(DE3). For protein production, the engineered strains were induced with 0.1 M isopropyl β‐D‐1‐thiogalactopyranoside (IPTG) at 16°C for 14 h. The resulting crude enzymes were purified using a HisTrap HP affinity column (GE Healthcare), and the purity of the fusion proteins was assessed via SDS‐PAGE analysis.

### 4.8 Characterization of Self‐Assembled Enzymatic Nanofibers

#### 4.8.1 Transmission Electron Microscopy

The morphological characteristics of the self‐assembled nanofibers were examined using TEM. Briefly, carbon‐coated copper grids were pre‐treated with air glow discharge for 1 min to enhance surface hydrophilicity. A 10 µL aliquot of the nanofiber suspension was deposited onto the grid, followed by immediate negative staining with 2% (w/v) phosphotungstic acid for 60 s. The prepared samples were subsequently imaged using an H‐7800 transmission electron microscope operating at an accelerating voltage of 80–120 kV [36, 45, 46].

#### 4.8.2 Fluorescence Analysis of Assembly

##### 4.8.2.1 ANS Fluorescence Assay

To characterize the structural evolution of the nanofibers, both surface hydrophobicity and fibrillation kinetics were monitored using extrinsic fluorescence probes. The changes in surface hydrophobicity during fibril growth were assessed using 8‐anilino‐1‐naphthalenesulfonic acid (ANS). Briefly, 17 µL of ANS was mixed with the protein sample and vortexed for 10 s. Fluorescence spectra were acquired using a spectrophotometer with an excitation wavelength of 370 nm and an emission scan range of 460–600 nm (slit widths: 5 nm excitation, 4 nm emission). Measurements were taken under light‐protected conditions at specific time points (0, 24, 48, 72, 120, and 168 h), and the maximum emission intensity was plotted to track hydrophobic exposure [38, 39].

##### 4.8.2.2 ThT Fluorescence Assay and Kinetic Fitting

Thioflavin T (ThT) fluorescence was employed to quantify amyloid fibril formation. The assay mixture consisted of 16.5 µL of protein sample, 475 µL of 10 mM Tris‐HCl buffer (pH 8.0), and 10 µL of ThT dye. After vortexing, fluorescence intensity was immediately recorded with excitation at 440 nm and emission at 484 nm (slit widths: 5 nm excitation, 6 nm emission) over the same 168 h period. The resulting time‐dependent fluorescence data were fitted to a sigmoidal Boltzmann model using Origin 10.0 software. The fitted parameters are summarized in Table S2 [41, 42, 43, 44].

#### 4.8.3 Tyndall‐Effect Observation

The colloidal character of the self‐assembled fusion‐enzyme nanofibers was examined using the Tyndall effect. Samples in assembly buffer were transferred to disposable 4.5 mL cuvettes with a 10 mm path length. A green laser (532 nm, <2 mW) was directed through each sample, and the visible light‐scattering path was recorded [37, 45].

### 4.9 Catalytic Characterization of Self‐Assembled Fusion‐Enzyme Nanofibers

The catalytic properties of the self‐assembled fusion‐enzyme nanofibers were evaluated across 30°C–60°C and pH 4.0‐9.0. Thermal and pH stability were assessed from residual activity after incubation under the indicated conditions (temperatures: 4°C and 37°C–65°C; pH 4.0–9.0). Substrate specificity was evaluated using 10 mM biogenic amines. Kinetic parameters were obtained from initial‐rate measurements at varying substrate concentrations using Lineweaver‐Burk plots. Activity was also measured with ethanol (0%–25% v/v) or NaCl (0%–20% w/v), with the additive‐free condition defined as 100% [25].

### 4.10 Magnetic Immobilization and Repeated‐Use Evaluation

Four magnetic carriers were evaluated. After vortex mixing, the materials were magnetically separated and washed with ultrapure water and triethanolamine buffer. They were activated with 5% glutaraldehyde at 25°C and 200 rpm for 3 h, then incubated with nanofiber suspensions at 20°C and 1000 rpm for approximately 18 h. Tris‐HCl buffer was used to terminate cross‐linking. Loading efficiency and capacity were calculated from protein concentrations before and after immobilization. Activity was measured across five reaction cycles (the initial cycle followed by four magnetic recovery cycles), with initial activity set to 100% [16, 17, 48, 49, 50, 51].

### 4.11 Catalytic Characterization of Magnetically Recyclable Enzymatic Nanofibers

Optimal pH and temperature of the magnetically recyclable enzymatic nanofiber composites were evaluated over pH 4.0–9.0 and 28°C–60°C. Stability was assessed from activity after 1 h incubation in buffers of different pH or at different temperatures. Effects of ethanol (0%–30% v/v) and NaCl (0%–16%) were evaluated with the additive‐free condition defined as 100% activity [27]. Ethanol stability was further assessed after 1 h incubation at the indicated concentrations [28].

### 4.12 Biogenic Amine Conversion in Fermented Foods and Simulated Wastewater

For fermented‐food tests, 50 µL of magnetically recyclable enzymatic nanofiber composite was added to 5 mL of sample, followed by HPLC determination of biogenic amines after 48 h. Controls received pretreated magnetic carrier without immobilized enzyme. The simulated wastewater contained each of the eight biogenic amines at 25 mg/L each (200 mg/L total); 50 µL of composite was added to 5 mL, and residual amines were measured after 48 h using the same carrier control [52, 53, 54].

### 4.13 Statistical Analysis

All experiments were performed in triplicate. Data were processed and analyzed using Origin Pro 9.0 software and GraphPad Prism 9.0. Statistical significance was evaluated by one‐way analysis of variance (ANOVA) followed by Tukey's multiple‐comparison test for the datasets presented in Figure 1d, Table 1, and Figure 7. A value of *p* < 0.05 was considered statistically significant. R 4.3.1 and JavaScript were employed for data calculation, processing, and graph generation.

## Author Contributions

S.L.: methodology, data curation, formal analysis, funding acquisition. Q.Y.: investigation, methodology, data curation, formal analysis, Writing – original draft. Q.Z.: Writing – review & editing. T.L.: data curation, investigation. X.H.: methodology, project administration. J.M.: formal analysis, funding acquisition.

## Conflicts of Interest

The authors declare no conflicts of interest.

## Supporting information




**Supporting File**: advs78579‐sup‐0001‐SuppMat.docx.

## Data Availability

The data that support the findings of this study are available from the corresponding author upon reasonable request.
