# Supplementary material for: Amyloid‐Guided Assembly of Magnetically Recyclable Enzymatic Nanofibers Toward Sustainable Biocatalysis
Source: Adv Sci (Weinh). 2026 Nov 5:e78579. Online ahead of print. doi: 10.1002/advs.78579 (PMC13735435; doi:10.1002/advs.78579)
Supplement: Supplementary file 1 [file ADVS-9999-e78579-s001.docx]

**Supporting Information**

**Amyloid-Guided Assembly of Magnetically Recyclable Enzymatic Nanofibers toward Sustainable Biocatalysis**

Shuangping Liu ^a,b,c^, Qilin Yang ^a^, Qian Zhang ^a,b^, Tiantian Liu ^a,b^, Xiao Han ^a,b,c^, Jian Mao ^a,b,c,*^

^a^State Key Laboratory of Food Science and Technology, School of Food Science and Technology, Jiangnan University, Wuxi, Jiangsu, 214122, China.

^b^Shaoxing Key Laboratory of Traditional Fermentation Food and Human Health, Jiangnan University (Shaoxing) Industrial Technology Research Institute, Shaoxing, Zhejiang, 312000, China

^c^National Engineering Research Center of *Huangjiu*, Zhejiang Guyuelongshan Shaoxing Wine Co., Ltd., Shaoxing, Zhejiang 312000, China.

*Corresponding author: Dr. Jian Mao.

E-mail: [maojian@jiangnan.edu.cn](mailto:maojian@jiangnan.edu.cn).

Address: School of Food Science and Technology, Jiangnan University, Wuxi, Jiangsu, 214122, China

**Figure S1.** DNA sequence alignment of PAO4 before and after codon optimization.

**Figure S2.** DNA sequence alignment of MAO7 before and after codon optimization.

**Figure S3.** Catalytic activity of linker-engineered fusion-enzyme variants following induction at different temperatures.

(A) *E. coli* BL21(DE3)/pET28a(+)-G1S1; (B) *E. coli* BL21(DE3)/pET28a(+)-G3S3; (C) *E. coli* BL21(DE3)/pET28a(+)-E1K1; (D) *E. coli* BL21(DE3)/pET28a(+)-E3K3.

**Figure S4.** Optimal reaction pH profiles of linker-engineered fusion-enzyme variants.

(A) G1S1; (B) G3S3; (C) E1K1; (D) E3K3.

**Figure S5.** pH stability profiles of linker-engineered fusion-enzyme variants.

(A) G1S1; (B) G3S3; (C) E1K1; (D) E3K3.

**Figure S6.** Optimal reaction temperature profiles of linker-engineered fusion-enzyme variants.

(A) G1S1; (B) G3S3; (C) E1K1; (D) E3K3.

**Figure S7.** Thermal stability profiles of linker-engineered fusion-enzyme variants.

(A) G1S1; (B) G3S3; (C) E1K1; (D) E3K3.

**Figure S8.** Ethanol-dependent relative activity of linker-engineered fusion-enzyme variants.

(A) G1S1; (B) G3S3; (C) E1K1; (D) E3K3.

**Figure S9.** Ramachandran plots of DPAO4 and DMAO7.

**Figure S10.** Representative snapshots from 100 ns molecular dynamics simulations of linker-engineered fusion-enzyme variants.

(A-C) G1S1 at 0, 50, and 100 ns; (D-F) G3S3 at 0, 50, and 100 ns; (G-I) E1K1 at 0, 50, and 100 ns; (J-L) E3K3 at 0, 50, and 100 ns.

**Figure S11.** Optimal reaction pH profiles of assembly-competent fusion enzymes bearing Sup35^1-61^ or Ure2^1-80^.

(A) URE2-E3K3; (B) SUP35-E3K3; (C) URE2-G3S3; (D) SUP35-G3S3.

**Figure S12.** pH stability profiles of assembly-competent fusion enzymes bearing Sup35^1-61^ or Ure2^1-80^.

(A) URE2-E3K3; (B) SUP35-E3K3; (C) URE2-G3S3; (D) SUP35-G3S3.

**Figure S13.** Optimal reaction temperature profiles of assembly-competent fusion enzymes bearing Sup35^1-61^ or Ure2^1-80^.

(A) URE2-E3K3; (B) SUP35-E3K3; (C) URE2-G3S3; (D) SUP35-G3S3.

**Figure S14.** Thermal stability profiles of assembly-competent fusion enzymes bearing Sup35^1-61^ or Ure2^1-80^.

(A) URE2-E3K3; (B) SUP35-E3K3; (C) URE2-G3S3; (D) SUP35-G3S3.

**Figure S15.** Effects of ethanol concentration on amyloid-guided enzymatic nanofibers.

(A) URE2-E3K3; (B) SUP35-E3K3; (C) URE2-G3S3; (D) SUP35-G3S3.

**Figure S16.** Effects of NaCl concentration on the relative activity of amyloid-guided enzymatic nanofibers.

(A) URE2-E3K3; (B) SUP35-E3K3; (C) URE2-G3S3; (D) SUP35-G3S3.

**Figure S17.** Relative activity of amyloid-guided enzymatic nanofibers after immobilization on different magnetic carriers.

Carrier a: 20 nm magnetic chitosan; carrier b: 100 nm magnetic chitosan; carrier c: MagZyme Si-Amine; carrier d: MagStart-Amine. For each construct, the activity obtained with carrier c was defined as 100%.

**Figure S18.** pH-dependent activity of magnetically recyclable enzymatic nanofiber composites.

(A) Fe_3_O_4_@SiO_2_-NH_2_@URE2-E3K3; (B) Fe_3_O_4_@SiO_2_-NH_2_@SUP35-E3K3.

**Figure S19.** Temperature-dependent activity of magnetically recyclable enzymatic nanofiber composites.

(A) Fe_3_O_4_@SiO_2_-NH_2_@URE2-E3K3; (B) Fe_3_O_4_@SiO_2_-NH_2_@SUP35-E3K3.

**Table S1.** Kinetic parameters of linker-engineered fusion-enzyme variants.

| **Enzyme** | **Parameter** | **HIS** | **TYR** | **PUT** | **CAD** | **PHE** | **TRY** | **SPE** | **SPD** |
| --- | --- | --- | --- | --- | --- | --- | --- | --- | --- |
| G1S1 | *V*max | 0.15 | 0.42 | 1.85 | 0.25 | 0.12 | 0.11 | 0.13 | 0.22 |
|  | *K*_m_ | 0.65 | 3.33 | 55.84 | 2.75 | 0.08 | 0.10 | 0.42 | 3.46 |
|  | *K*_i_ | - | - | - | - | 582.30 | 130.50 | - | - |
|  | *k*cat | 2.49 | 7.01 | 30.58 | 4.13 | 1.91 | 1.77 | 2.17 | 3.58 |
|  | *k*cat/*K*_m_ | 3.80 | 2.11 | 0.55 | 1.50 | 23.59 | 17.11 | 5.23 | 1.04 |
| G3S3 | *V*max | 1.95 | 1.80 | 0.79 | 4.69 | 2.02 | 1.12 | 0.46 | 0.14 |
|  | *K*_m_ | 1.00 | 0.04 | 16.03 | 13.91 | 0.06 | 0.03 | 8.44 | 0.88 |
|  | *K*_i_ | - | 12.64 | 36.33 | 4.69 | 171.60 | 74.08 | 408.00 | - |
|  | *k*cat | 32.23 | 29.69 | 13.06 | 77.34 | 33.38 | 18.47 | 7.63 | 2.27 |
|  | *k*cat/*K*_m_ | 32.23 | 753.46 | 0.81 | 5.56 | 562.95 | 731.88 | 0.90 | 2.59 |
| E1K1 | *V*max | 0.19 | 0.45 | 1.52 | 0.34 | 0.18 | 0.17 | 0.18 | 0.25 |
|  | *K*_m_ | 0.65 | 2.53 | 28.67 | 2.63 | 1.34 | 1.22 | 0.55 | 1.71 |
|  | *K*_i_ | - | - | - | - | 76.73 | 45.60 | - | - |
|  | *k*cat | 3.18 | 7.47 | 25.10 | 5.53 | 3.05 | 2.85 | 2.99 | 4.05 |
|  | *k*cat/*K*_m_ | 4.91 | 2.95 | 0.88 | 2.10 | 2.28 | 2.34 | 5.41 | 2.37 |
| E3K3 | *V*max | 0.22 | 0.74 | 1.08 | 0.34 | 0.17 | 0.18 | 0.16 | 0.27 |
|  | *K*_m_ | 0.50 | 4.39 | 10.26 | 2.26 | 0.35 | 0.89 | 0.16 | 1.48 |
|  | *K*_i_ | - | 53.35 | - | - | 132.10 | 57.88 | - | - |
|  | *k*cat | 3.59 | 12.24 | 17.81 | 5.62 | 2.73 | 2.91 | 2.62 | 4.44 |
|  | *k*cat/*K*_m_ | 7.24 | 2.79 | 1.74 | 2.49 | 7.86 | 3.27 | 16.22 | 3.01 |

Units: *K*_m_ and *K*_i_, mM; *V*_max_, μmol/(L·min); *k*_cat_, s^−1^; *k*_cat_/*K*_m_, L/(s·mmol).

**Table S2.** Boltzmann-model parameters for amyloid-guided assembly kinetics of four fusion-enzyme variants.

| Construct | ymax | y0 | t1/2 | *k* | Lag time (h) | R² |
| --- | --- | --- | --- | --- | --- | --- |
| URE2-G3S3 | 53.73±5.58 | 136.63±1.06 | 35.34±3.06 | -0.05952 | 68.94 | 0.99298 |
| URE2-E3K3 | 63.67±5.26 | 142.34±1.27 | 43.56±3.54 | -0.04941 | 84.04 | 0.99265 |
| SUP35-G3S3 | 25.80±39.91 | 226.12±1.44 | 9.05±7.60 | -0.05504 | 45.39 | 0.99329 |
| SUP35-E3K3 | 48.60±10.09 | 240.55±3.18 | 44.77±2.51 | -0.06702 | 74.61 | 0.9907 |

**Table S3.** EDS elemental composition of magnetic carriers before and after enzymatic nanofiber immobilization.

|  | Fe₃O₄@SiO₂-NH₂ | | Fe₃O₄@SiO₂-NH₂@URE2-E3K3 | | Fe₃O₄@SiO₂-NH₂@SUP35-E3K3 | |
| --- | --- | --- | --- | --- | --- | --- |
| **Element** | **Weight %** | **Atomic %** | **Weight %** | **Atomic %** | **Weight %** | **Atomic %** |
| C | 11.11 | 21.52 | **15.79** | **29.77** | **15.39** | **27.99** |
| N | 0.14 | 0.24 | **0.75** | **1.21** | **1.06** | **1.65** |
| O | 39.19 | 56.97 | 34.22 | 48.43 | 38.18 | 52.11 |
| Si | 1.31 | 1.08 | 1.24 | 1.00 | 1.1 | 0.85 |
| P | 0 | 0 | **0.19** | **0.14** | **0.06** | **0.04** |
| S | 0.28 | 0.21 | 0.27 | 0.19 | 0.26 | 0.18 |
| Fe | 47.97 | 19.98 | 47.54 | 19.27 | 43.96 | 17.19 |
| Total |  |  | 100.00 | 100.00 | 100.00 | 100.00 |

**Table S4.** Sequence information for linkers and primers.

| Name | Sequence (5′-3′) |
| --- | --- |
| Linker |  |
| G1S1 | GGTGGCGGTGGCTCG |
| G3S3 | GGAGGAGGTGGAAGCGGAGGAGGAGGAAGCGGAGGAGGAGGAAGC |
| E1K1 | GAGGAAGAGGAAAAGAAAAAGAAA |
| E3K3 | GAAGCTGCGGCAAAAGAAGCAGCGGCTAAAGAAGCGGCGGCAAAA |
| Primer |  |
| G1S1-F1 | GTGCCGCGCGGCAGCCATATGACCATGCATCCGCTGGAACCGCTGA |
| G1S1-R1 | TCCATACTTCCTCCTCCTCCGCTTTCGCAGCAATGGCTGCCGGTGCTCGGGTT |
| G1S1-F2 | GGCAGCCATTGCTGCGAAAGCGGAGGAGGAGGAAGTATGGATCGCTATGATGCGGT |
| G1S1-R2 | TTGTCGACGGAGCTCgaattcTTTTTCCGCCGCCAGAATTTCCGCCGCGGTGCGTTT |
| G3S3-F1 | GTGCCGCGCGGCAGCCATATGACCATGCATCCGCTGGAACCGCTGA |
| G3S3-R1 | GCTTTCGCAGCAATGGCTGCCGGTGCTCGGGTTCGGCGGCACATCCAGG |
| G3S3-F2 | GGCAGCCATTGCTGCGAAAGCGGAGGAGGTGGAAGCGGAGGAGGAGGAAGCGGAGGAGGAGGAAGCATGGATCGCTATGATGCGGT |
| G3S3-R2 | TTGTCGACGGAGCTCgaattcTTTTTCCGCCGCCAGAATTTCCGCCGCGGTGCGTTT |
| E1K1-F1 | GTGCCGCGCGGCAGCCATATGACCATGCATCCGCTGGAACCGCTGA |
| E1K1-R1 | TCCATCTTGGCGGCGGCTTCGCTTTCGCAGCAATGGCTGCC |
| E1K1-F2 | GGCAGCCATTGCTGCGAAAGCGAAGCCGCCGCCAAGATGGATCGCTATGATGCGGT |
| E1K1-R2 | TTGTCGACGGAGCTCgaattcTTTTTCCGCCGCCAGAATTTCCGCCGCGGTGCGTTT |
| E3K3-F1 | GTGCCGCGCGGCAGCCATATGACCATGCATCCGCTGGAACCGCTGA |
| E3K3-R1 | GCTTTCGCAGCAATGGCTGCCGGTGCTCGGGTTCGGCGG |
| E3K3-F2 | CGGCAGCCATTGCTGCGAAAGCGAAGCCGCCGCCAAGGAAGCCGCCGCCAAGGAAGCCGCCGCCAAGATGGATCGCTATGATGCGGT |
| E3K3-R2 | TTGTCGACGGAGCTCgaattcTTTTTCCGCCGCCAGAATTTCCGCCGCGGTGCGTTT |
| OL-F | CGCGGCAGCCATATGGCTAGCATGACCATGCATCCGCTGGAACCGCTGAGCGCCGCGGAAGTG |
| OL-R | TTGTCGACGGAGCTCgaattcTTTTTCCGCCGCCAGAATTTCCGCCGCGGTGCGTTTGCCGCTTTCAAT |
